# Resolutions on the Death of Dr. Henry Fisher

**Published:** 1893-09

**Authors:** 


### Obituary.

### RESOLUTIONS ON THE DEATH OF DR. HENRY FISHER.

WHEREAS, in the sudden death of Dr. Henry Fisher the St. Louis Dental Society has lost one of its oldest and best members, a faithful and trustworthy officer of many years, and one who had the highest interest of the Society and profession at heart; be it

*Resolved*, That our heartfelt sympathy be tendered to the bereaved family; and

*Resolved*, That a copy of this preamble and resolutions be sent to the family of the deceased, and also to the daily press and leading dental journals for publication.

W. H. EAMES.
WM. N. MORRISON.
J. B. NEWBY.

ST. LOUIS, MO., August 3, 1893.

